# Endogenous erythropoietin has immunoregulatory functions that limit the expression of autoimmune kidney disease in mice

**DOI:** 10.3389/fimmu.2023.1195662

**Published:** 2023-07-13

**Authors:** Sofia Bin, Chiara Cantarelli, Julian K. Horwitz, Micaela Gentile, Manuel Alfredo Podestà, Gaetano La Manna, Peter S. Heeger, Paolo Cravedi

**Affiliations:** ^1^ Precision Immunology Institute, Translational Transplant Research Center, Icahn School of Medicine at Mount Sinai, New York, NY, United States; ^2^ Nephrology, Dialysis and Renal Transplant Unit, Istituto di Ricovero e Cura a Carattere Scientifico (IRCCS) - Azienda Ospedaliero-Universitaria di Bologna, Bologna, Italy; ^3^ Centro Interdipartimentale di Ricerca Industriale (CIRI) Scienze della Vita e Tecnologie per la Salute - Alma Mater Studiorum Università di Bologna, Bologna, Italy; ^4^ Dipartimento di Medicina e Chirurgia, Università di Parma, Unità Operativa (UO) Nefrologia, Azienda Ospedaliero-Universitaria di Parma, Parma, Italy; ^5^ Ronald Reagan Medical Center, University of California at Los Angeles (UCLA), Los Angeles, CA, United States; ^6^ Renal Division, Department of Medicine, Transplantation Research Center, Brigham and Women’s Hospital, Harvard Medical School, Boston, MA, United States; ^7^ Department of Medicine, Comprehensive Transplant Center, Cedars-Sinai Medical Center, Los Angeles, CA, United States

**Keywords:** germinal center B cell, TFH, TfR, systemic lupus erythematosus, erythropoietin

## Abstract

**Background:**

Administration of recombinant erythropoietin (EPO), a kidney-produced hormone with erythropoietic functions, has been shown to have multiple immunoregulatory effects in mice and humans, but whether physiological levels of EPO regulate immune function *in vivo* has not been previously evaluated.

**Methods:**

We generated mice in which we could downregulate EPO production using a doxycycline (DOX)-inducible, EPO-specific silencing RNA (shEPOrtTA^POS^), and we crossed them with B6.MRL-Fas^lpr^/J mice that develop spontaneous lupus. We treated these B6.MRL/lpr shEPOrtTA^POS^ with DOX and serially measured anti-dsDNA antibodies, analyzed immune subsets by flow cytometry, and evaluated clinical signs of disease activity over 6 months of age in B6.MRL/lpr shEPOrtTA^POS^ and in congenic shEPOrtTA^NEG^ controls.

**Results:**

In B6.MRL/lpr mice, *Epo* downregulation augmented anti-dsDNA autoantibody levels and increased disease severity and percentages of germinal center B cells compared with controls. It also increased intracellular levels of IL-6 and MCP-1 in macrophages.

**Discussion:**

Our data in a murine model of lupus document that endogenous EPO reduces T- and B-cell activation and autoantibody production, supporting the conclusion that EPO physiologically acts as a counterregulatory mechanism to control immune homeostasis.

## Introduction

Erythropoietin (EPO), which is produced predominantly by kidney perivascular interstitial fibroblasts, fetal liver cells, and monocytes/macrophages (1), stimulates hematopoiesis through ligating a homodimeric erythropoietin receptor (EPOR) on red blood cell (RBC) precursors that initiates a JAK2/STAT5-dependent signaling cascade and prevents their apoptosis (2). Emerging evidence from our research group among several others showed that administration of recombinant EPO can induce pleiotropic immunoregulatory properties, distinct from EPO’s ability to stimulate RBC production (1). In this context, EPO therapy given at doses used for the treatment of anemia inhibits proliferation of naïve and memory murine and human CD4^+^ and CD8^+^ T cells (3); prevents CD4^+^ T_H1_, T_H17_, and T_FH_ cell differentiation and expansion (3–5); facilitates regulatory T-cell (Treg) generation and expansion (6, 7); and promotes differentiation of regulatory macrophages (7). Through these various mechanisms, EPO administration reduces the severity of murine experimental autoimmune encephalomyelitis (EAE), limits development of murine interstitial nephritis, prevents clinical expression of multiple models of murine lupus nephritis (LN), and promotes murine heart transplant survival (4, 6, 8). Findings from these studies in mice were corroborated by evidence that recombinant EPO increases the number and function of circulating Treg in patients with autoimmune hepatitis (9) and in patients with chronic kidney disease (6).

Despite this significant body of work, it remains unknown whether physiological levels of endogenously produced EPO function beyond stimulating hematopoiesis and contribute to immune homeostasis. To address this issue, we generated a mouse model in which EPO production can be downregulated by a doxycycline (DOX)-driven promoter that transcribes an EPO-binding, small hairpin RNA (shRNA). Using this system, we analyzed the effects of *Epo* downregulation on a murine, immune dependent kidney disease model and assessed associated mechanisms.

## Materials and methods

### Murine *Epo* knockdown model

A shRNA guide sequence targeting *Epo* was selected based on *in vitro Epo* knockdown experiments in mouse kidney cell lines. Prediction of the potency and specificity of shRNA to target inducible knockdown of mouse erythropoietin was performed using two distinct proprietary algorithms by Mirimus Inc. (Brooklyn, NY). The potency of the top candidates was then evaluated using Mirimus’ sensor assay, ranking each shRNA tested by activity. Briefly, each shRNA was cloned into its LPEK retroviral vector, a process that requires oligo library assembly and synthesis followed by PCR-based cloning and sequence verification. Mirimus used this proprietary assay to assess the function of each predicted shRNA sequence in an unbiased manner. The target site of each shRNA sequence is cloned into the 3′UTR of a fluorescent reporter, and each shRNA was measured for its ability to silence the reporter in comparison with a number of validated control shRNAs. A tetracycline-responsive, shRNA guide strand sequence used in this study was 5′ TTTTCTGCCTCCTTGGCCTCTA 3′. A XhoI/EcoRI fragment containing both the miR30 scaffold and the passenger/guide strands of the Epo.199 shRNA was cloned into tetracycline-responsive, miR30-based shRNA targeting vectors, as previously described (10). Embryonic stem cells (ESCs) were targeted and screened as described previously (11, 12) and mice generated by tetraploid embryo complementation. The transgene utilizes the CAG promoter to drive the expression of the reverse tetracycline-controlled transactivator (rtTA3). In bitransgenic founder mice containing both TRE-shRNA and a rtTA, DOX feeding activates the TRE that induces shRNA expression in all tissues.

To evaluate the role of endogenous EPO in a model of systemic lupus erythematosus (SLE), shEPO^+/+^ CAGs-rtTA3^+/-^ (shEPOrtTA^POS^) mice were then bred at Icahn School of Medicine at Mount Sinai with B6.MRL-Fas^lpr^/J mice (The Jackson Laboratory, Bar Harbor, ME) that develop spontaneous lupus, to obtain B6.MRL/lpr shEPOrtTA^POS^ and B6.MRL/lpr shEPOrtTA^NEG^ controls. These mice with a B6 background (*H2^b^*) have a milder disease course than those on an MRL/MpJ (*H2^k^*) background (13), allowing us to test the hypothesis that the absence of EPO worsens disease severity.

### Experimental lupus model

At 1 month of age, female B6.MRL/lpr shEPOrtTA^POS^ and B6.MRL/lpr shEPOrtTA^NEG^ controls were fed DOX (for up to 5 months) to induce a specific shRNA that binds to and degrades *Epo* mRNA, lowering its transcription and reducing EPO protein production. At 145 days after DOX treatment, animals were euthanized and kidneys and spleen harvested.

All the experiments were performed in littermates or animals maintained in the same room and/or co‐housed within the same cages for at least 2 weeks to limit potential influences of different microbiomes.

The murine studies were performed at the Icahn School of Medicine under IACUC-approved protocol number 2017-0273, originally approved in May 2017, renewed for May 2020 through May 2023.

### Quantitative real-time PCR

Total RNA was isolated from whole mouse kidneys using TRIzol (Thermo Fisher Scientific, Waltham, MA). cDNA was synthesized using reverse transcription reagent (Thermo Fisher Scientific). Real-time PCR assays were performed using the TaqMan Universal PCR Master Mix and primer set for *Epo* (Mm01202755_m1; Thermo Fisher Scientific). Relative expression was normalized to that of the housekeeping gene *Gapdh* (Mm99999915_g1; Thermo Fisher Scientific).

### Serum EPO concentrations and hematocrit quantification

Blood samples were collected by puncture of the submandibular vein.

Serum EPO concentrations were determined using Mouse Erythropoietin ELISA Kit (#ab270893, Abcam, Waltham, MA) following the manufacturer’s instructions.

To measure hematocrit levels, samples were spun down for 60 s in conventional hematocrit capillary tubes using HemataStat II™ Hematocrit Centrifuge (EKF Diagnostics Inc., San Antonio, TX).

### Anti-dsDNA antibodies

ELISA was performed on serum samples and anti-dsDNA autoantibody concentrations (unit/mL) measured using a commercial quantitative mouse anti-dsDNA IgG-specific ELISA kit (Alpha Diagnostic Intl. Inc., San Antonio, TX).

### Urinary albumin and creatinine measurement

Urine samples were obtained from individual mice through gentle restrain. Urinary creatinine was quantified using commercial kits from Cayman Chemical (Ann Harbor, MI). Urinary albumin was determined using a commercial assay from Bethyl Laboratories Inc. (Montgomery, TX). Albuminuria was expressed as the ratio of urinary albumin to creatinine.

### Assessment of skin severity score and survival

Inflammatory skin lesions on the dorsum of the neck, ears, and forehead were scored basing on a scale of 0–3 as previously described (14): 0 = no visible skin changes, 1 = minimal hair loss with redness and a few scattered lesions, 2 = redness, scabbing, and hair loss with a small area of involvement, and 3 = ulcerations with an extensive area of involvement.

Animal survival in the experimental and control group was monitored and the results reported in a Kaplan–Meier survival curve.

### Renal histology

Mice were anesthetized through i.p. injection of a solution of sterile ketamine (200–300 mg/kg) and xylazine (20–30 mg/kg) in PBS and perfused with 4% paraformaldehyde *via* intracardiac injection at a rate of 8–10 mL/min. Periodic acid–Schiff (PAS) staining was performed on paraffin-embedded (10%) kidney sections (3 μm thick), and imaging brightfield was acquired on a widefield microscope (Zeiss Axio Imager Z2M).

### Flow cytometry

Standard approaches for surface and intracellular staining were used, as previously published (4, 5). For surface staining, the following antibodies were used: biotinylated anti-CXCR5 (BD Pharmingen, San Diego, CA) followed by Pacific Blue streptavidin (Thermo Fisher Scientific); PerCP-Cy5.5-anti-TCRb (clone H57-597), PE-Cy7-anti-PD1 (clone RMP1-30), BV510-anti-CD4 (clone GK1.5), and Pacific Blue-anti-CD8a (clone 53-6.7) (BioLegend, San Diego, CA); FITC-anti-CD25 (clone 7D4), Pacific Blue-anti-B220 (clone RA 3-6 B2), APC-anti-FAS (clone Jo2), and FITC-anti-GL7 (clone GL7) (BD Pharmingen); APC-Cy7-anti-IgD (clone 11-26c), PE-Cy7-anti-IgM (clone eB121-15F9), PerCP-Cy5.5-anti-CD11b (clone M1/70), and PE-anti-F4/80 (clone BMB) (Thermo Fisher Scientific); and PE-Cy7-anti-CD4 (clone GK1.5) (Tonbo Biosciences, San Diego, CA). After cell permeabilization using the eBioscience™ Foxp3/Transcription Factor Staining Buffer Set (Thermo Fisher Scientific), intracellular staining was performed using the following antibodies: FITC-anti-Foxp3, APC-anti-Foxp3 (clone FJK-16S), FITC-anti-IL-6 (clone MP520F3), and FITC-anti-MCP-1 (clone 2H5) (Thermo Fisher Scientific); Pacific Blue-anti-TNF-α (clone MP6-XT22), APC-anti-IL-10 (clone JES5-16E3), PE-Cy7-anti-IL-2 (clone JES6-5H4), BV421-anti-IFN-γ (clone XMG1.2), and PE-Cy7-anti-IL-4 (clone 11B11) (BioLegend).

Data were acquired (at least 10,000 to 100,000 events, in most cases >100,000 events) on a three-laser Canto II flow cytometer (BD Biosciences) and analyzed using FlowJo (https://www.flowjo.com) software.

### Statistical analyses

Two-group comparisons and multiple comparisons among treatment groups were analyzed by unpaired t test, Mann–Whitney test, or Multiple Mann–Whitney test. For survival curve comparisons, we used log-rank (Mantel-Cox). *P* values < 0.05 were considered significant. All statistical analyses were performed using GraphPad Prism (version 8 for Windows, GraphPad Software, Inc.).

## Results

### Validation of the shEPOrtTA mouse phenotype

**Figure 1:**
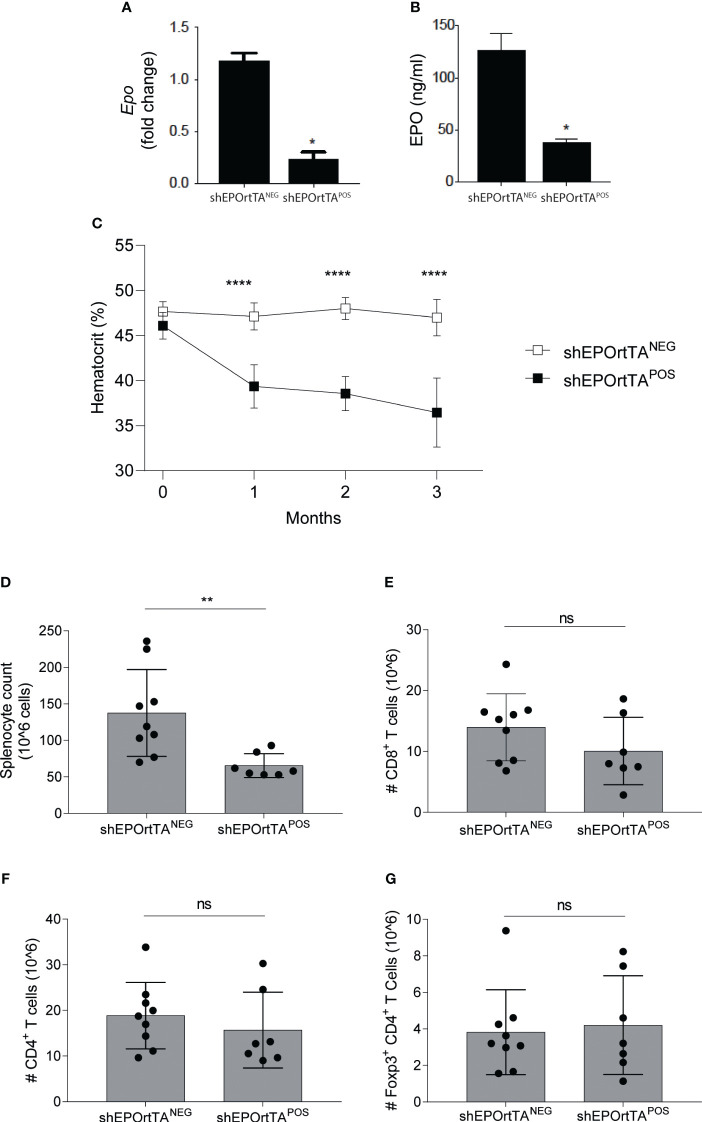
Characterization of EPO knockdown mice. **(A)** Renal *Epo* mRNA expression and **(B)** serum EPO levels at month 3 after DOX initiation and **(C)** hematocrit changes at months 0 to 3 after DOX initiation in shEPOrtTA^POS^ (n=9) and shEPOrtTA^NEG^ controls (n=7). **(D)** Total splenocytes, **(E)** CD8^+^ T cells, **(F)** CD4^+^ T cells, and **(G)** Treg in the spleen at 3 months after DOX initiation. Data were analyzed using **(A, B)** t test, *P < 0.05; **(C)** Multiple Mann–Whitney test, ****P <0.00001; **(D–G)** Mann–Whitney U test, *P <0.01, ns: not significant.

To verify that DOX feeding reduced systemic EPO in shEPOrtTA^POS^ mice compared with shEPOrtTA^NEG^ controls, we treated both cohorts with DOX-containing chow. One month later, analyses showed significantly lower transcripts of *Epo* by RT-PCR in the kidney and reduced serum protein expression in shEPOrtTA^POS^ animals (**Figures 1A, B**). Continued DOX feeding led to a progressively lower hematocrit over a 3-month follow-up period (**Figure 1C**). After 3 months of DOX food administration, we observed fewer total spleen cells compared with controls (**Figure 1D**), a potential consequence of reduced reticulocyte production, without significant change in the numbers of CD8^+^ and CD4^+^ T cells or CD4^+^CD25^+^Foxp3^+^ Treg (**Figures 1E-G**).

### Endogenous EPO reduces disease severity in murine lupus

We measured renal *Epo* gene expression in WT and B6.MRL/lpr mice, and no significant difference was detected, although B6.MRL/lpr mice trended toward higher levels (**Supplementary Figure 1**).

**Figure 2:**
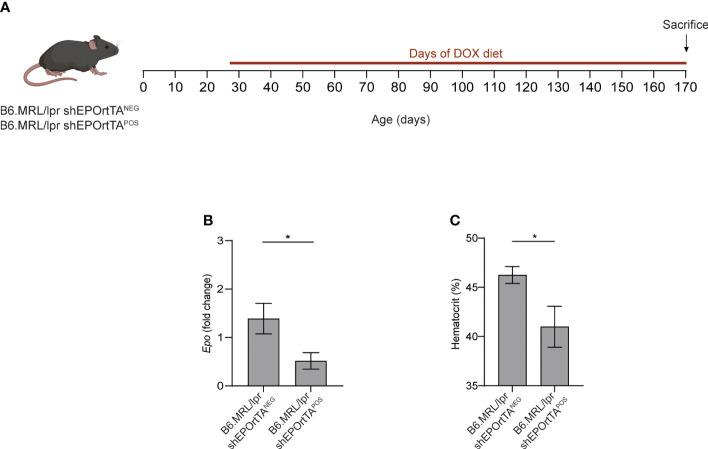
Validation of inducible *Epo* downregulation in B6.MRL/lpr mice. **(A)** Experimental design. **(B)** *Epo* mRNA expression in kidney from B6.MRL/lpr shEPOrtTA^POS^ (n=23) and shEPOrtTA^NEG^ controls (n=15). **(C)** Hematocrit levels in B6.MRL/lpr shEPOrtTA^POS^ and shEPOrtTA^NEG^ mice at 2 months after DOX initiation. t test; *P < 0.05.

We next fed both B6.MRL/lpr shEPOrtTA^POS^ and B6.MRL/lpr shEPOrtTA^NEG^ controls with DOX food beginning at 1 month of age (**Figure 2A**), verifying that DOX food reduced both *Epo* gene expression in the kidney and hematocrit in the B6.MRL/lpr shEPOrtTA^POS^ mice (**Figures 2B, C**).

**Figure 3:**
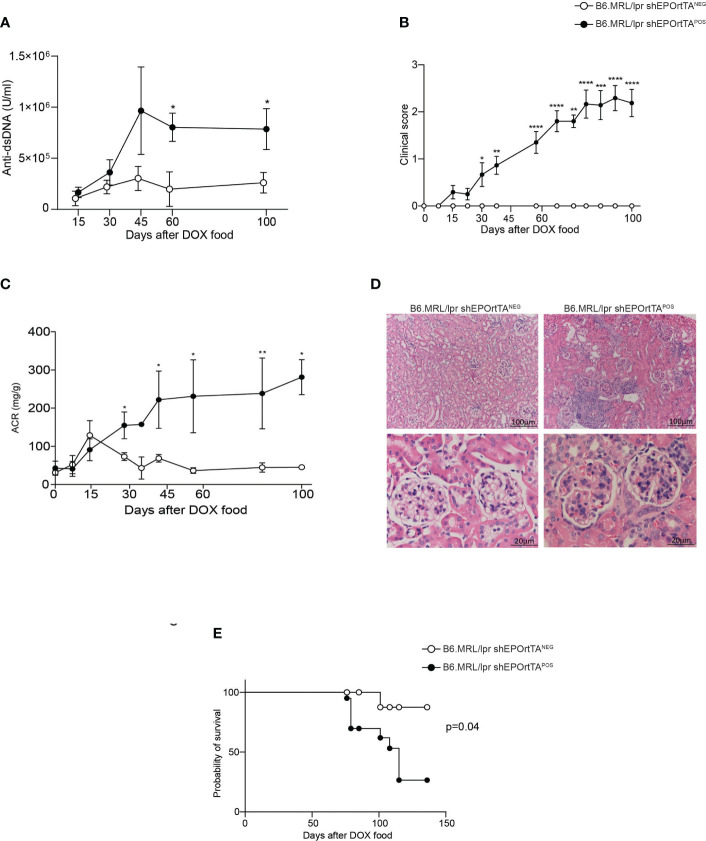
Downregulating endogenous EPO production increases disease severity and shortens survival in a murine lupus model. **(A)** Anti-dsDNA autoantibody levels in B6.MRL/lpr shEPOrtTA^POS^ (n=12) and shEPOrtTA^NEG^ (n=9). **(B)** Clinical score of skin lesions developed in B6.MRL/lpr shEPOrtTA^POS^ (n=23) compared with shEPOrtTA^NEG^ controls (n=14). **(C)** Albumin-to-creatinine ratio (ACR) in B6.MRL/lpr shEPOrtTA^POS^ (n=19) and shEPOrtTA^NEG^ (n=15) mice. **(D)** Representative images of PAS staining of kidney tissue at sacrifice; scale bars: 20–100 μm; magnification 10×–40×. Multiple Mann–Whitney test; *P < 0.05; **P < 0.01; ***P < 0.001; ****P < 0.0001 between B6.MRL/lpr shEPOrtTA^POS^ and shEPOrtTA^NEG^. **(E)** Survival (%) of B6.MRL/lpr shEPOrtTA^POS^ (n=20) and shEPOrtTA^NEG^ (n=14) mice; log-rank (Mantel–Cox) test; *P < 0.05.

Serum analyses showed significantly more anti-dsDNA autoantibodies in B6.MRL/lpr shEPOrtTA^POS^ mice compared with controls, beginning 2 months after starting DOX administration (**Figure 3A**). The DOX-treated B6.MRL/lpr shEPOrtTA^POS^ animals developed skin lesions consistent with cutaneous lupus with the same kinetics as the detection of anti-dsDNA autoantibodies (**Figure 3B**). In contrast, no skin lesions appeared in the control animals (**Figure 3B**). B6.MRL/lpr shEPOrtTA^POS^ mice but not the controls progressively developed albuminuria as well as glomerulonephritis and interstitial kidney infiltrates over 3 months following initiation of DOX-containing chow (**Figures 3C, D**). Follow-up of additional cohorts showed reduced survival of the DOX-treated B6.MRL/lpr shEPOrtTA^POS^ animals compared with controls (**Figure 3E**).

### *Epo* downregulation augments GC B cells

**Figure 4:**
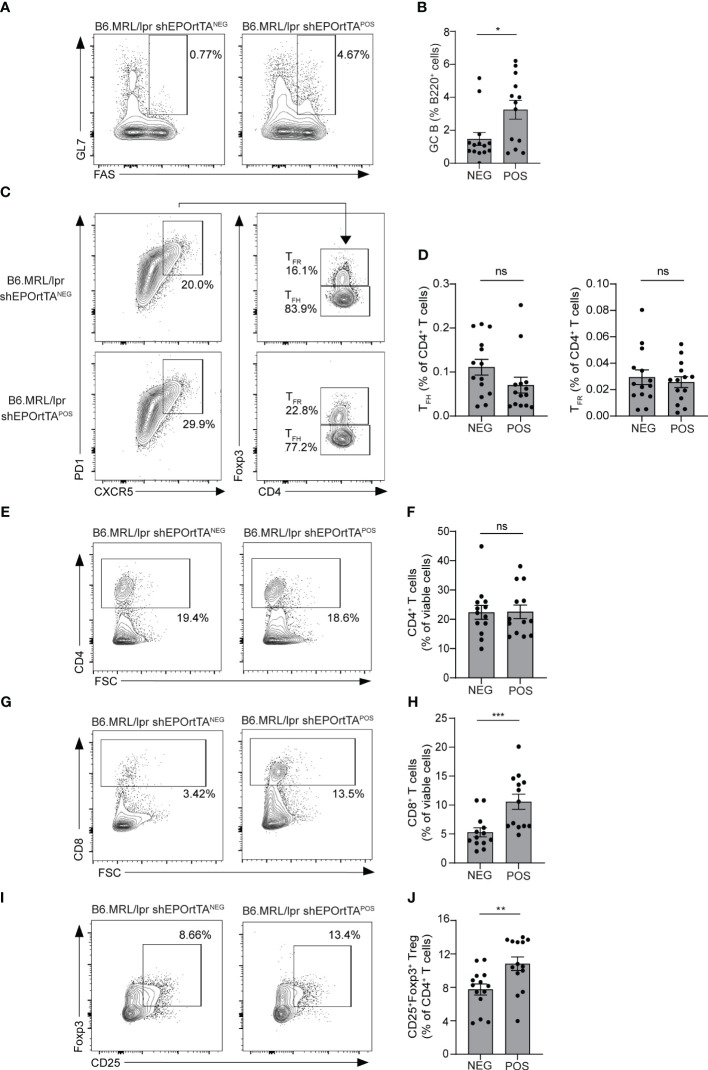
Effects of endogenous EPO downregulation on GC B-cell and T-cell subsets. **(A, B)** Representative plots and data quantification for splenic B220^+^Fas^+^GL7^+^ GC B cells; **(C, D)** CD4^+^PD1^+^CXCR5^+^Foxp3^-^ T_FH_ and CD4^+^PD1^+^CXCR5^+^Foxp3^+^ T_FR_; **(E, F)** total CD4^+^ T cells and **(G, H)** CD8^+^ T cells; **(I, J)** CD4^+^CD25^+^Foxp3^+^ Treg in B6.MRL/lpr shEPOrtTA^POS^ (n=14) and shEPOrtTA^NEG^ (n=14) at day 145 after DOX food initiation. Mann–Whitney U test; ns, not significant, *P < 0.05; **P < 0.01; ***P < 0.001 between B6.MRL/lpr shEPOrtTA^POS^ and shEPOrtTA^NEG^ groups.

Our prior data indicate that EPO inhibits auto- and allo-antibody production primarily by reducing T-cell-dependent germinal centers (GC) (5, 7). We used flow cytometry to quantify splenic B220^+^Fas^+^GL7^+^ GC B cells at the time of sacrifice in DOX-fed B6.MRL/lpr shEPOrtTA^POS^ and shEPOrtTA^NEG^ controls. Consistent with our previous findings in which EPO-treated mice showed reduced GC B cells, analysis of spleen cells from DOX-treated B6.MRL/lpr shEPOrtTA^POS^ showed higher frequencies of B220^+^Fas^+^GL7^+^ GC B cells *vs*. controls (**Figures 4A, B**).

Frequencies of PD1^+^CXCR5^+^Foxp3^-^ T_FH_ and of PD1^+^CXCR5^+^Foxp3^+^ T_FR_ did not differ between B6.MRL/lpr shEPOrtTA^POS^ and shEPOrtTA^NEG^ animals (**Figures 4C, D**), as well as percentages of CD4^+^ T cells (**Figures 4E, F**), whereas percentages of CD8^+^ T cells and CD4^+^CD25^+^Foxp3^+^ Treg significantly increased in B6.MRL/lpr shEPOrtTA^POS^ (**Figures 4G-J**).

### *Epo* downregulation promotes macrophage activation

**Figure 5:**
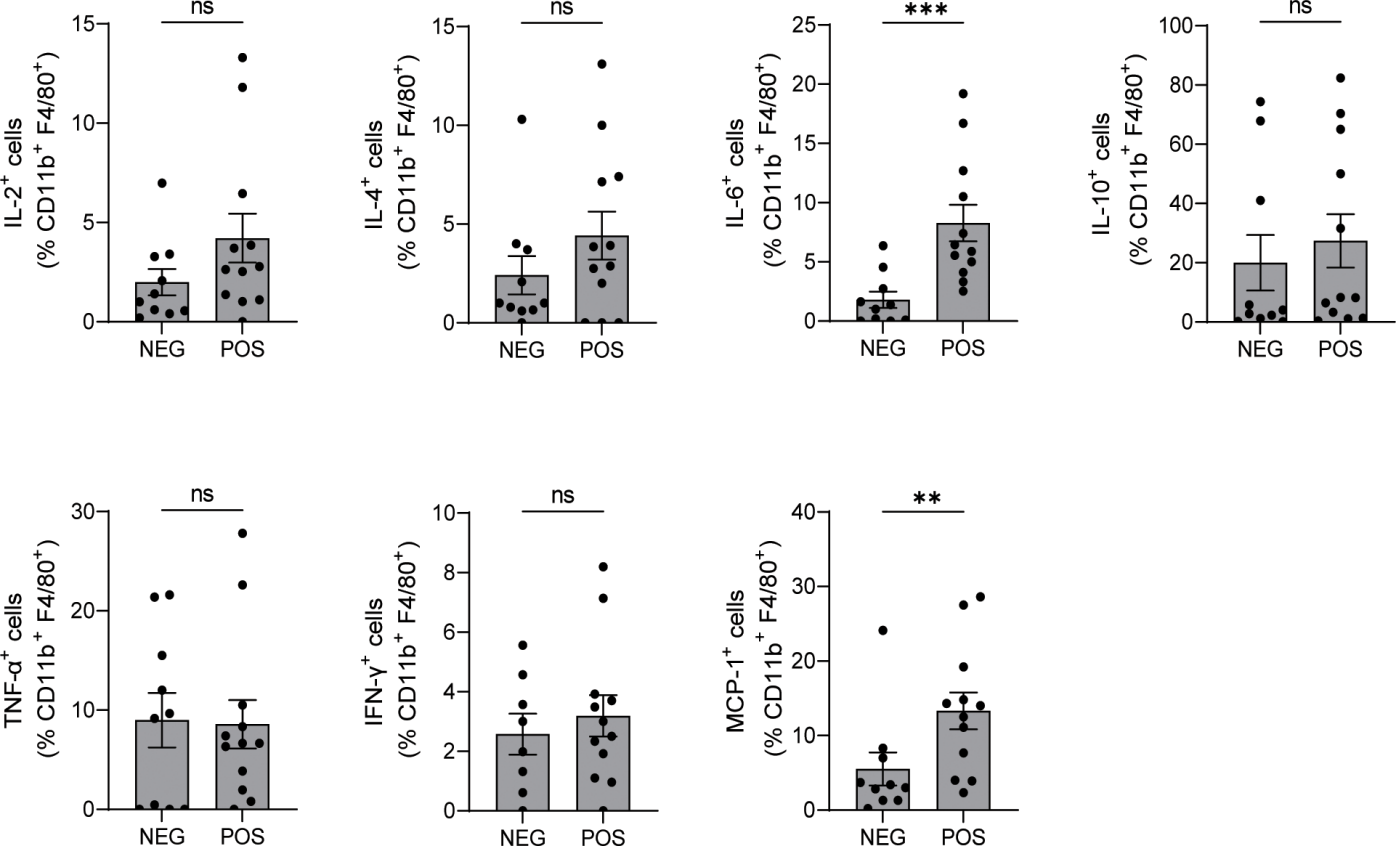
Downregulating endogenous EPO production increases IL-6 and MCP-1 production in macrophages. Intracellular cytokine production in CD11b^+^ splenic macrophages isolated from B6.MRL/lpr shEPOrtTA^POS^ (n=12) and shEPOrtTA^NEG^ controls (n=10) at day 145 after DOX food initiation (flow cytometry). Mann–Whitney U test; ns, not significant, **P < 0.01; ***P < 0.001 between B6.MRL/lpr shEPOrtTA^POS^ and shEPOrtTA^NEG^ groups.

Macrophages produce EPO (15), and previous work by others showed that autocrine EPO signaling in macrophages, activated by molecular signals of dying cells and hypoxia (15, 16), is required for the development of immune tolerance, clearance of cellular debris, and resolution of inflammation (15, 16). To test the effects of *in vivo Epo* downregulation on splenic macrophage activation, we first verified that macrophages from shEPOrtTA^POS^ mice had no *Epo* gene expression (**Supplementary Figure 2**). Next, we analyzed the macrophage expression of intracellular cytokines in the same B6.MRL/lpr shEPOrtTA^POS^ and shEPOrtTA^NEG^ mice used for the immune-phenotyping analyses above. These assays showed significantly higher levels of pro-inflammatory mediators IL-6 and MCP-1 in B6.MRL/lpr shEPOrtTA^POS^ animals *vs*. controls, whereas no significant differences were detected in IL-2, IL-4, IL-10, TNF-α, and IFN-γ levels between groups (**Figure 5** and **Supplementary Figure 3**).

## Discussion

Using a murine model of kidney disease in which *Epo* RNA can be reduced using a shRNA, we newly demonstrate that physiological levels of EPO exert immunomodulating effects that counteract inflammation. Spontaneous development of lupus-like autoimmunity and glomerulonephritis commonly occur in MRL-Fas^lpr^ mice over 6–8 months of age. While congenic B6.MRL/lpr mice do not routinely develop kidney disease, they develop autoantibodies and lymphoid hyperplasia and permit mechanistic analysis. As downregulating EPO production promoted the development of LN in these mice, it is reasonable to speculate that EPO production represents one of the mechanisms that contribute to B6 strain resistance to LN. Importantly, lack of endogenous EPO significantly reduced mouse survival, suggesting that EPO plays a major role in limiting immune responses.

Our prior findings showed that high doses of recombinant EPO counteract B-cell maturation and autoantibody formation by inhibiting T_FH_ activation *via* a STAT5-dependent mechanism (5). However, the EPO concentrations reached by such approach are not physiological. The present finding that *Epo* downregulation resulted in increased autoantibody production and GC B-cell expansion suggests that EPO exerts immune effects even at physiological concentrations, but the definition of underlying molecular mechanisms will require additional studies.

Intriguingly, we found that *Epo* downregulation increased CD8^+^ T cells in B6.MRL/lpr mice, but not in WT animals. Therefore, it is tempting to speculate that immunomodulatory mechanisms of EPO become more apparent during inflammatory disease, consistent with our prior data showing that recombinant EPO directly inhibits effector T-cell proliferation (5, 6). We also previously showed that EPO promotes Treg induction through a TGF-β-mediated mechanism (6). Therefore, we hypothesized that *Epo* downregulation would have resulted in lower Treg frequencies. Contrary to our prediction, shEPOrtTA^POS^ mice had higher, not lower, Treg percentages, possibly as a compensatory mechanism driven by the overall uncontrolled autoimmune response. The lack of functional data on Treg suppressive capacity does not allow to exclude the alternative hypothesis that, when *Epo* is downregulated, Treg function is impaired.

Defective macrophage activity is a key element in SLE pathogenesis and correlates with disease severity (17). Previous studies by others have shown that EPO is required for effective clearance of apoptotic bodies by monocytes/macrophages (15). Herein, we found increased levels of IL-6 and MCP-1 in mice with *Epo* downregulation, consistent with the observation that animals with conditional deletion of *Epor* in myeloid cells have higher levels of these cytokines (15, 16). Overall, these data suggest that macrophage produced EPO signals through EPOR in an autocrine fashion to prevent macrophage activation. This mechanism, together with EPO inhibitory effects on T cells, concurs to support a critical role of EPO in preventing progression of lupus and, potentially, other autoimmune diseases (18).

We acknowledge that the B6.MRL-Fas^lpr^/J mouse model of lupus nephritis does not fully recapitulate the features of human disease (19). However, as patients with SLE often present functional autoantibodies against EPO or EPOR that associate with faster disease progression (20–23), we speculate that impaired EPO production or signaling further unleashes the autoimmune response in affected individuals.

In sum, our data indicate that endogenous EPO is not implicated in maintaining peripheral tolerance, as mice with *Epo* downregulation do not spontaneously show signs of systemic disease. However, when a disease process is initiated, EPO seems to play the counterregulatory mechanisms that are important in maintaining immune homeostasis.

## Data availability statement

The raw data supporting the conclusions of this article will be made available by the authors, without undue reservation.

## Ethics statement

The animal study was reviewed and approved by Icahn School of Medicine.

## Author contributions

SB, CC, JH, MG performed the experiments, contributed to experimental design, analyzed data and wrote the manuscript. MP and GL helped in writing the manuscript. PH and PC designed and oversaw the study, analyzed data, and wrote the manuscript. All authors read and approved the final manuscript. All authors have approved the final article and agree with its publication.
