# Supplementary figures and images for: Endogenous erythropoietin has immunoregulatory functions that limit the expression of autoimmune kidney disease in mice
Source: Front Immunol. 2023 Jul 13;14:1195662. doi: 10.3389/fimmu.2023.1195662 (PMC10381939; doi:10.3389/fimmu.2023.1195662)

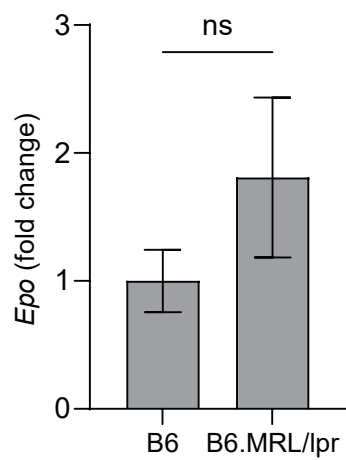

**Supplementary Figure 1.** Renal *Epo* mRNA expression in B6 (n=3) and B6.MRL/lpr (n=3).  
t test; ns: not significant

Supplement: Supplementary file 1 [file DataSheet_1.pdf]
