# Supplementary material for: Endogenous erythropoietin has immunoregulatory functions that limit the expression of autoimmune kidney disease in mice
Source: Front Immunol. 2023 Jul 13;14:1195662. doi: 10.3389/fimmu.2023.1195662 (PMC10381939; doi:10.3389/fimmu.2023.1195662)
Supplement: Supplementary file 2 [file DataSheet_2.pdf]

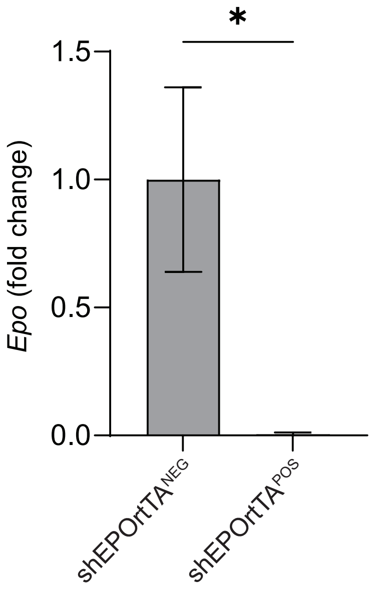

Supplementary Figure 2. *Epo* mRNA expression in splenic macrophages in shEPOrtTA^{NEG} (n=4) and shEPOrtTA^{NEG} (n=4). t test; *P <0.05.
