# Supplementary material for: Endogenous erythropoietin has immunoregulatory functions that limit the expression of autoimmune kidney disease in mice
Source: Front Immunol. 2023 Jul 13;14:1195662. doi: 10.3389/fimmu.2023.1195662 (PMC10381939; doi:10.3389/fimmu.2023.1195662)
Supplement: Supplementary file 3 [file DataSheet_3.pdf]

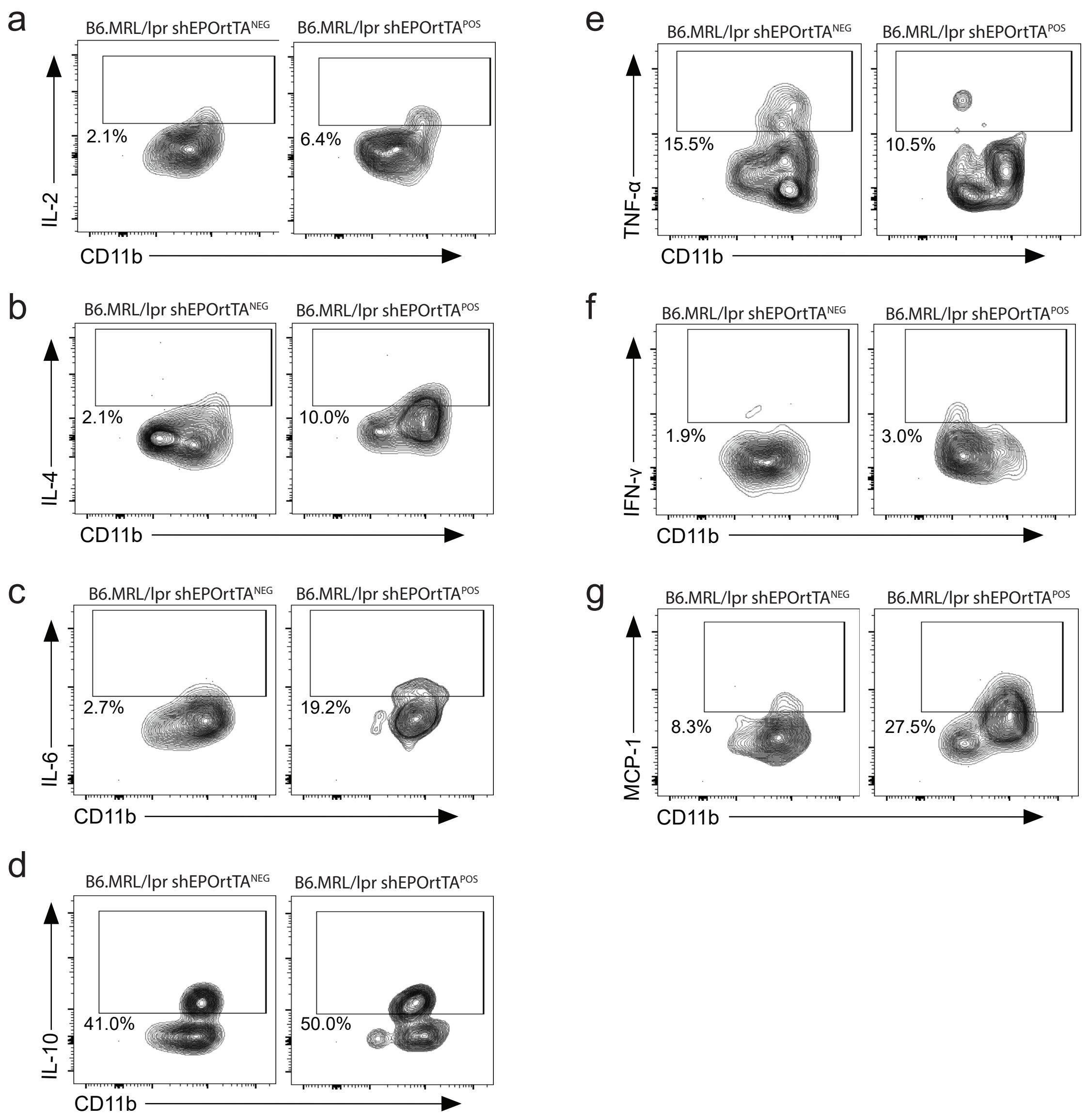

Supplementary Figure 3. Representative plots for (a) IL-2, (b) IL-4, (c) IL-6, (d) IL-10, (e) TNF-α, (f) IFN-γ , and (g) MCP-1 in $CD11b^{+}$ macrophages from B6.MRL/lpr $shEPOrtTA^{POS}$ and $shEPOrtTA^{NEG}$ mice.
